# Nutrient conditions determine the strength of herbivore‐mediated stabilizing feedbacks in barrens

**DOI:** 10.1002/ece3.9929

**Published:** 2023-03-21

**Authors:** Laia Illa‐López, Àlex Aubach‐Masip, Teresa Alcoverro, Giulia Ceccherelli, Luigi Piazzi, Periklis Kleitou, Jorge Santamaría, Jana Verdura, Neus Sanmartí, Elvira Mayol, Xavi Buñuel, Mario Minguito‐Frutos, Fabio Bulleri, Jordi Boada

**Affiliations:** ^1^ Institut de Ciències del Mar (ICM_CSIC) Passeig Marítim de la Barceloneta Barcelona Spain; ^2^ Centre d'Estudis Avançats de Blanes (CEAB‐CSIC) Blanes Spain; ^3^ Departament de Biologia Evolutiva Ecologia i Ciències Ambientals Facultat de Biologia Universitat de Barcelona Barcelona Spain; ^4^ Nature Conservation Foundation Mysore Karnataka India; ^5^ Dipartimento di Scienze Chimiche, Fisiche Matematiche e Naturali Università di Sassari Sassari Italy; ^6^ Marine and Environmental Research (MER) Lab Limassol Cyprus; ^7^ Université Côte d'Azur, CNRS UMR 7035 ECOSEAS Nice France; ^8^ Federative Research Institute ‐ Marine Resources Université Côte d'Azur Nice France; ^9^ Institut Mediterrani d'Estudis Avançats (IMEDEA‐CSIC) Esporles Spain; ^10^ Dipartimento di Biologia Università di Pisa CoNISMa Pisa Italy; ^11^ Laboratorie d'Océanographie de Villefranche‐sur‐Mer CNRS Sorbonne Université Villefranche sur mer France

**Keywords:** alternative stable states, environmental conditions, feedbacks, herbivory, limpets, marine forests

## Abstract

Abiotic environmental conditions can significantly influence the way species interact. In particular, plant–herbivore interactions can be substantially dependent on temperature and nutrients. The overall product of these relationships is critical for the fate and stability of vegetated ecosystems like marine forests. The last few decades have seen a rapid spread of barrens on temperate rocky reefs mainly as a result of overgrazing. The ecological feedbacks that characterize the barren state involve a different set of interactions than those occurring in vegetated habitats. Reversing these trends requires a proper understanding of the novel feedbacks and the conditions under which they operate. Here, we explored the role of a secondary herbivore in reinforcing the stability of barrens formed by sea urchin overgrazing under different nutrient conditions. Combining comparative and experimental studies in two Mediterranean regions characterized by contrasting nutrient conditions, we assessed: (i) if the creation of barren areas enhances limpet abundance, (ii) the size‐specific grazing impact by limpets, and (iii) the ability of limpets alone to maintain barrens. Our results show that urchin overgrazing enhanced limpet abundance. The effects of limpet grazing varied with nutrient conditions, being up to five times more intense under oligotrophic conditions. Limpets were able to maintain barrens in the absence of sea urchins only under low‐nutrient conditions, enhancing the stability of the depauperate state. Overall, our study suggests a greater vulnerability of subtidal forests in oligotrophic regions of the Mediterranean and demonstrates the importance of environment conditions in regulating feedbacks mediated by plant–herbivore interactions.

## Graphical abstract

Abiotic environmental conditions can significantly influence plant–herbivore interactions. We evaluate the capacity of limpet grazing to act as reinforcing feedback of barrens. We demonstrate the critical effect of nutrients in determining limpet–algae interactions which were found to maintain barrens stable under oligotrophic conditions.

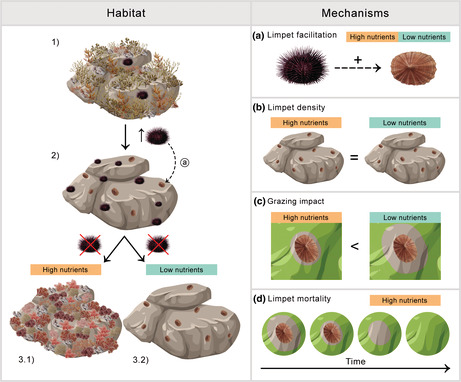

## 1 INTRODUCTION

Abrupt regime shifts are common in many natural systems. Beyond a threshold of stability, rapidly accelerating feedbacks kick in destabilizing ecosystems, eventually shifting the system into an alternate state that is typically less diverse and functionally deteriorated (Scheffer et al., 2001). Once the shift has occurred, these systems can be maintained in the altered state by reinforcing feedbacks (Conversi et al., 2015; Nyström et al., 2012). The strength of feedback mechanisms determines the way ecosystems change and their persistence in time (Nyström et al., 2012). In the absence of reinforcing feedbacks, or when destabilizing feedbacks are stronger, ecosystems shift between different states tracking different trajectories based on contingent conditions (Scheffer et al., 2001). For instance, in the Serengeti‐Mara, bushfires can promote the transition from woodlands to grasslands that, once established, are actively maintained by the grazing of mega‐herbivores (Dublin et al., 1990). Woodland recovery is promoted by a major decline in herbivore abundance. Therefore, understanding the role that destabilizing or reinforcing feedbacks play in determining ecosystem state is essential to manage ecosystem characterized by non‐linear behaviors.

Ecosystems may suffer small or dramatic reorganization (changes in the community structure) when biotic and abiotic conditions are altered due to natural or anthropogenic disturbances. Conversi et al. (2015) defined ecological regime shifts as abrupt ecosystem re‐organizations, encompassing multiple variables, which are persistent in time and involve key structural species from a wide range of trophic levels and ecological roles. This includes organisms such as top predators and key grazers (Estes & Palmisano, 1974; Ripple et al., 2014; Wolf et al., 2007), resource providers, such as primary producers, decomposers and nitrogen‐fixing species (Reid et al., 1998; Scheffer et al., 1993; Stinca et al., 2015), and ecosystem engineers (Knowlton, 1992). This moves away from the traditional debate on the prevalence of top‐down versus bottom‐up processes as drivers of regime shifts to a more nuanced unpacking of the interaction between trophic/biological and physical/environmental drivers. For instance, environmental drivers such as ocean warming and marine heatwaves have led to a major restructuring of plant–herbivore interactions, as tropical herbivores expand to temperate areas and discover new resources (Vergés et al., 2016; Vergés, Steinberg, et al., 2014). These novel interactions can be catastrophic, leading in some cases to the complete collapse of structural species (Ling et al., 2009).

In the marine realm, strong feedbacks also mediate the resilience of pelagic and benthic ecosystems. For example, increased nutrient loading and intensive overfishing of herbivorous fishes in coral reefs allow fleshy macroalgae to overgrow coral structures (Hughes, 1994; Knowlton, 1992). Similarly, anthropogenic nutrient loads in semi‐enclosed marine systems, like estuaries, gulfs, and coastal lagoons, stimulates phytoplankton production which reduces the amount of light reaching the bottom, and compromises the stability of seagrass meadows (Lindegren et al., 2012; Petersen et al., 2008). As in terrestrial landscapes, grazing is one of the main drivers controlling the composition and distribution of marine benthic communities and is often an important agent of ecosystem collapses (Bakker, 1998; Bulleri et al., 1999; Scheibling et al., 2009). Abiotic factors, such as temperature and nutrients, determine the strength of species interactions, including consumer–resource relationships, which effect scale up to the ecosystem level (Boada et al., 2017; Pagès et al., 2018).

A commonly observed shift in shallow subtidal ecosystems along temperate coastlines is the loss of macroalgal forests such as kelps and other canopy‐forming algae as a result of sea urchin outbreaks or range‐extending non‐native species (Estes & Palmisano, 1974; Filbee‐Dexter & Scheibling, 2014; Ling et al., 2009; Vergés, Tomas, et al., 2014). The formation of barren grounds implies a drastic reduction in diversity, habitat structure, and productivity of rocky reefs (Filbee‐Dexter & Scheibling, 2014; Konar & Estes, 2003; Pinna et al., 2020; Steneck et al., 2002). They are often linked to a weakening of reinforcing feedback mechanisms that sustain the stability of marine forests, typically when the density of predatory species declines or are overexploited (see Ling et al., 2009; Sala & Zabala, 1996; Shears & Babcock, 2002). At the same time, new processes can contribute to establishing the barren state through, for instance, the enhancement of juvenile sea urchin survival (Filbee‐Dexter & Scheibling, 2014) and a reduced recruitment of shrub‐forming macroalgae (Bulleri et al., 2002).

Although sea urchins are the main agents responsible of barren formation (Ling et al., 2015), other herbivore species may play a key role enhancing barren stability once they have established (Bulleri et al., 2002; Creese, 1982; Scheibling et al., 2009). Limpets are often very abundant in shallow‐water barren areas (Andrew & Underwood, 1993; Bulleri et al., 1999). In the Mediterranean, tidal ranges are small (tens of centimeters) limpets belonging to the genus *Patella* are common across a vertical range, encompassing both intertidal and shallow subtidal rocky habitats (Bulleri et al., 1999; Piazzi et al., 2016; Prusina et al., 2014). More specifically, they can be abundant from low‐shore levels (i.e., 0.5 m above mean lower low water; MLLW), down to about 10 m depths (Benedetti‐Cecchi et al., 2001; Bulleri et al., 2000). They play a key role in regulating macroalgal distribution and abundance by feeding on microalgae and macroalgal spores and juvenile stages associated with epilithic biofilms (Jenkins & Hartnoll, 2001). While several studies demonstrated the importance of limpets in contributing to maintain urchin barrens (Andrew, 1991; Piazzi et al., 2016), it is still unclear under which biotic and abiotic circumstances their stabilizing effects are effective and how they vary at regional scales. Assessing the dependence of limpet grazing effects over environmental conditions requires to further explore the strength of limpet‐seaweed interaction under different local environmental settings (e.g., nutrients or temperature; Boada et al., 2017; Kriegisch et al., 2020; Nyström et al., 2012; Pagès et al., 2018) and to determine whether the grazing impact is size‐specific, as found for other mesograzers (Pessarrodona et al., 2019). Indeed, the effectiveness of limpets in controlling macroalgae re‐colonization depends upon background nutrient levels and may decrease with increasing productivity (Jenkins & Hartnoll, 2001; Piazzi et al., 2016). The key question is under which circumstances this process may result in a significant reinforcing feedback of degraded barrens even after sea urchins are removed. Specifically, under elevated nutrient conditions, the rapid growth of the algal cover could limit the ability of limpets to maintain free space, either bare or colonized by encrusting coralline algae (Underwood & Jernakoff, 1981).

In the present study, we examine whether nutrient conditions define the capacity of grazing by limpet of different sizes to act as a stabilizing mechanism of urchin barrens on Mediterranean rocky reefs. We assessed (i) whether the formation of sea urchin barrens enhances limpet abundance, (ii) size‐specific limpet grazing effects on algae, and (iii) whether the ability of limpets to maintain the barren state, in the absence of sea urchins, varies under contrasting nutrient conditions. For this, we (i) conducted field surveys in regions characterized by contrasting nutrient conditions to assess the relationships between algal cover, sea urchins and limpets, (ii) quantified limpet size‐specific grazing impact by comparing overgrazed areas around limpets (halos), under both high‐ and low‐nutrient conditions, and (iii) performed sea urchin removal experiments in large barren areas, under contrasting nutrient conditions.

## 2 MATERIALS AND METHODS

### 2.1 Study site and experimental setup

Mediterranean shallow rocky reefs are characterized by photophilic algal communities, dominated (when environmental conditions are optimal) by canopies of Fucales and other erect algae including some genera of Dictyotales and Sphacelariales. They create a highly structured and diverse habitat that facilitates recruitment and provides shelter to fishes and invertebrates (Bulleri et al., 2002; Sala et al., 2012; Sales & Ballesteros, 2009). Unfortunately, compounded human disturbances are compromising the persistence of Mediterranean marine forests, including overgrazing by sea urchins and non‐native species (i.e., rabbitfish), pollution and marine heatwaves (de Caralt et al., 2020; Sala et al., 2011; Verdura et al., 2021). Sea urchins are common on shallow rocky reefs and are the key herbivores regulating the structure of macroalgal communities and, following population outbreaks, they can trigger the shift to the barren state (Boada et al., 2018; Pinnegar et al., 2000; Sala et al., 2012). In particular, *Paracentrotus lividus* (Lamarck, 1816) and *Arbacia lixula* (Linneaus, 1758) are the dominant species on Mediterranean rocky reefs. Other herbivores, such as the limpets *Patella caerulea* (Linnaeus, 1758) and *Patella aspera* (Röding, 1798) can be common in shallow subtidal rock habitats and it has been suggested that they may play a role in maintaining barren states (Bulleri et al., 2002; Mauro et al., 2003; Piazzi et al., 2016). Nutrient conditions are highly variable within the Mediterranean basin, ranging from the very oligotrophic Eastern Mediterranean to the nutrient‐enriched continental shores of the Western basin. These differences can determine the vulnerability of marine forests to grazing (Boada et al., 2017).

**FIGURE 1:**
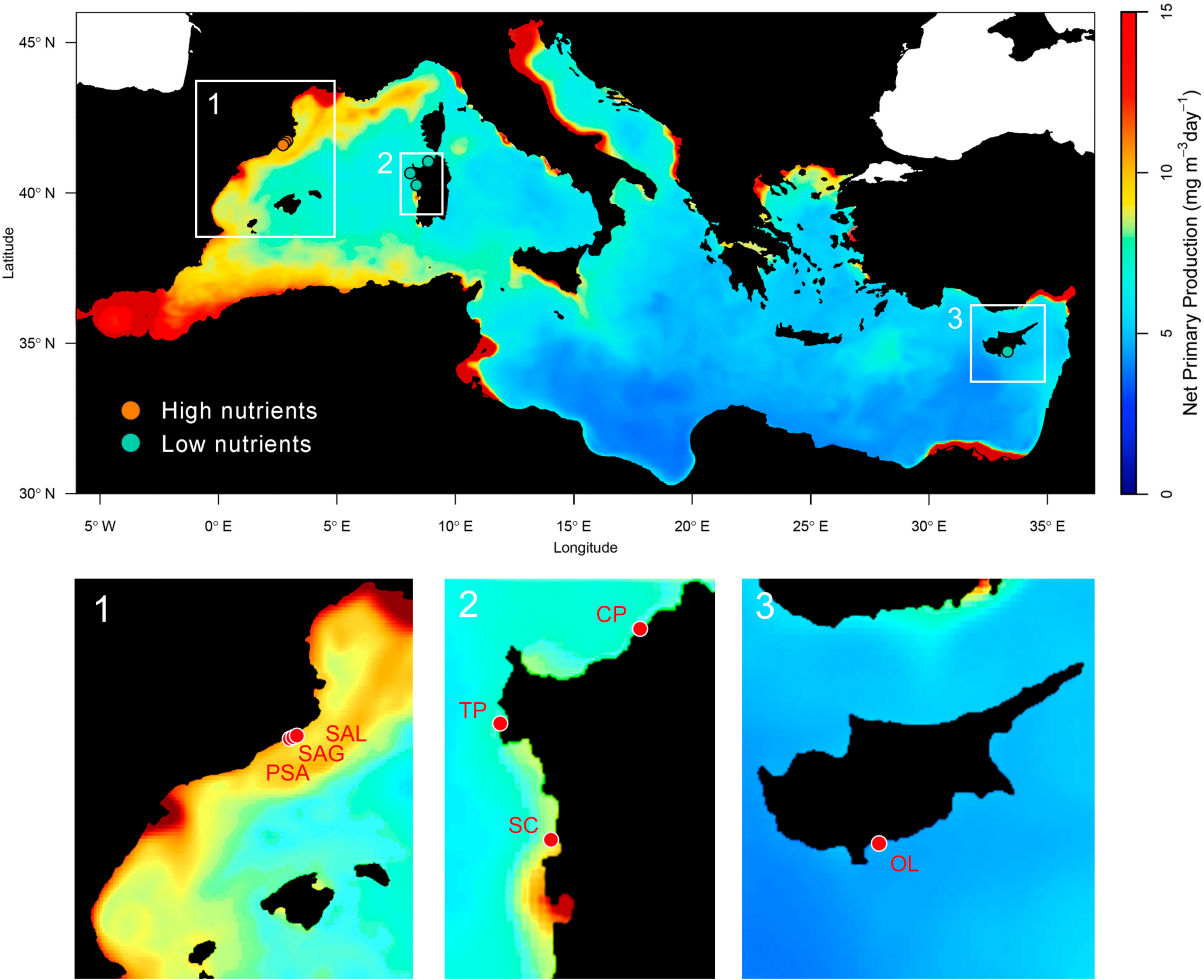
Map of the study locations (Mediterranean Sea). High‐nutrient regime surveys were conducted in (PSA) Punta Santa Anna (41.67286°N 2.80211°E), (SAG) S'Agulla Garbí (41.68192°N 2.81511°E) and (SAL) S'Agulla Llevant (41.68242°N 2.81725°E) in the Catalan coast (1), Spain (NW Mediterranean). Low‐nutrient regime surveys were conducted in (CP) Costa Paradiso (41.04980°N 8.93686°E), (TP) Torre Porticciolo (40.64287°N 8.18720°E) and (SC) Santa Caterina (40.10620°N 8.48231°E) in the west coast of Sardinia (2), Italy (NW Mediterranean) and in (OL) Old Limassol (34.70693°N 33.13508°E) in the south coast of Cyprus (3, Levantine Sea). Orange and turquoise dots in the upper panel represent the position of the surveyed sites within both high‐ and low‐nutrient conditions, respectively. Mean annual surface NPP map was produced using 2019 monthly series of the MedBFM model system obtained from CMEM's *Mediterranean Sea Biochemistry Analysis and Forecast* metadata (E.U. Copernicus Marine Service Information). Annual mean values were calculated from 2019 monthly averages using SeaDAS software from NASA (version 8.0.0).

We selected a total of seven sites, three in a high‐nutrient region (the Catalan coast, Spain) and four in low‐nutrient regions (Italy and Cyprus, Figure 1). Locations are known to be different in nutrient conditions based on satellite imagery data, as well as nutrient analyses of *Posidonia oceanica* tissues, a very integrative measurement of nutrient availability (see Boada et al., 2017; Roca et al., 2015). The surveyed sites had similar benthic assemblages and were separated by 2 to hundreds of kilometers. For all sites, we measured algal cover, sea urchin and limpet densities. All surveys were conducted by SCUBA at depths between 1 and 7 m. Additionally, to evaluate the effect of limpets in maintaining barrens in the absence of sea urchins, we performed manipulative experiments along the Catalan coast and in Sardinia.

#### 2.1.1 Limpet facilitation by sea urchins

To explore if sea urchins facilitate limpets by providing suitable surfaces for attachment (i.e., without erect macroalgae), we estimated total algal cover, as well as sea urchin and limpet abundance. At each site, we randomly placed a series of quadrats on the reef (40 × 40 cm and 50 × 50 cm in the Catalan coast and Sardinia, respectively) and took photos using an underwater camera (*n* = 50 photos per site, with a total of 289 photoquadrats: *n* = 125 in the high‐nutrient region and *n* = 164 in the low‐nutrient region). Photos were analyzed in the laboratory, to estimate algal cover, sea urchin and limpet densities within each quadrat. We established three vegetation groups as a proxy for the community state: (i) encrusting coralline algae or bare rock (i.e., barren), (ii) ephemeral algae forming an horizontal compact mat (i.e., turf), and (iii) a structured stratum with arborescent shrub‐forming algae displaying a more complex three‐dimensional structure. We assessed the percentage of surface covered by each of the three strata types using *ImageJ* software (National Institutes of Health).

#### 2.1.2 Limpet grazing impact

To test how the effects of grazing impact of limpets vary across regions, we estimated their size (i.e., shell area) and grazing marks (i.e., halos) from the photoquadrats (see above). A caliper or a ruler was carefully placed on the reef to be visible in photoquadrats and used as a scale reference to calculate limpet size. Thereafter, each limpet was assigned to one of six different size classes, according to shell area (i.e., [0–2 cm^2^], [2–4 cm^2^], [4–6 cm^2^], [6–8 cm^2^], [8–10 cm^2^] and [>10 cm^2^]). Halos result from herbivores overgrazing effect (i.e., consumption exceeding algae growth, see Pessarrodona et al., 2019 and Figure S1) representing a clear mark of limpets feeding activity. After analyzing data from photoquadrats (see above) we identified that some limpet sizes‐classes were underrepresented and, therefore, we increased the number of replicates by taking additional measurements of limpets‐halos in both high‐ and low‐nutrient conditions, obtaining a complete range of the different sizes. Then, to infer the grazing impact between contrasting nutrient conditions and limpet size, we estimated the ratio halo area to limpet shell area (i.e., grazing impact = halo area/limpet shell area).

#### 2.1.3 Sea urchin removal experiment

To assess if limpets have a differential role in promoting the persistence of barrens under contrasting nutrient conditions, we experimentally removed sea urchins in two study regions. We selected a total of six barren areas in each region and completely removed sea urchins from three of them while maintaining the other three as controls (i.e., sea urchins were left at natural densities). In each barren, we assessed the total algal cover – here referred as macroalgal recovery considering both turf and shrub‐forming cover – using the same procedure described above (see section 2.1.1). We did this at the beginning of the experiment, before removing the sea urchins, and, thereafter, every four to 6 months in the high‐nutrient region (for 1 year) and every 2 months in the low‐nutrient region (for a total of 6 months). We also recorded changes in limpet densities between sampling times to test if the recovery of algal cover limits the ability of limpets to persist in experimental barrens. Additionally, we measured the abundance of limpet halos without individuals as a proxy of limpet mortality (Figure S1). These clear grazing marks on the rock without any limpet are easily recognizable and distinguishable from other halos (e.g., sea urchins). The fact that limpets are not present indicates either mortality or migration but given a survival strategy based on spending the shortest time possible away from the home scar and the low mobility of these species, the probability of overestimation when attributing this proxy to mortality is very low (Jenkins & Hartnoll, 2001).

### 2.2 Data treatment and statistical analysis

#### 2.2.1 Monitoring study

We used a generalized linear mixed model (GLMM) to explore the effect of barren cover (%) and nutrient regime on limpet density (ind/m^2^). “Limpet density” was introduced as the response variable in the model, and “barren cover” and “Nutrient condition” (two levels: high nutrients and low nutrients) as the explanatory variables (fixed factors). “Site” (three levels; i.e., the survey localities within each region) was included as a random factor. To examine the differences in total limpet density (ind/m^2^) within each size class and in limpet grazing impact between nutrient conditions, we also performed a set of GLMM. In these cases, we set “Nutrient condition” as a fixed factor (two levels) and “site” as a random factor (three levels). Finally, to test for differential grazing impact among limpet size classes, we used a GLMM in which “halo area” was set as the response variable and “shell area” and “Nutrient condition” as an explanatory variable. Again “site” was set as a random factor. All GLMMs were fitted with a negative binomial distribution to cope with overdispersion (Zuur et al., 2009).

#### 2.2.2 Removal experiment

We assessed the development of the “algal cover” (%), “limpet density” (ind/m^2^) and the “number of halos without limpet” per square meter after removing sea urchins as response variables, by using a series of GLMM fitted a Poisson distribution across the entire time series. “Time” was introduced as a fixed factor (four levels – the survey times) and “Site” was set as a random factor (three levels). For multiple comparisons within “Time”, we applied the Tukey test using the “lsmeans” function from the “*lsmeans”* package (Lenth, 2016).

All analyses were computed in R (R Development Core Team 2021) using the “*lme4*” and *“glmmMTB”* packages (Bates et al., 2015). We used the DHARMa package in R to assess model fitting (Brooks et al., 2017; Hartig, 2022). We used the “*ggplot2*” package (Wickham, 2016) for graphical representations.

## 3 RESULTS

### 3.1 Limpet facilitation by sea urchins

**FIGURE 2:**
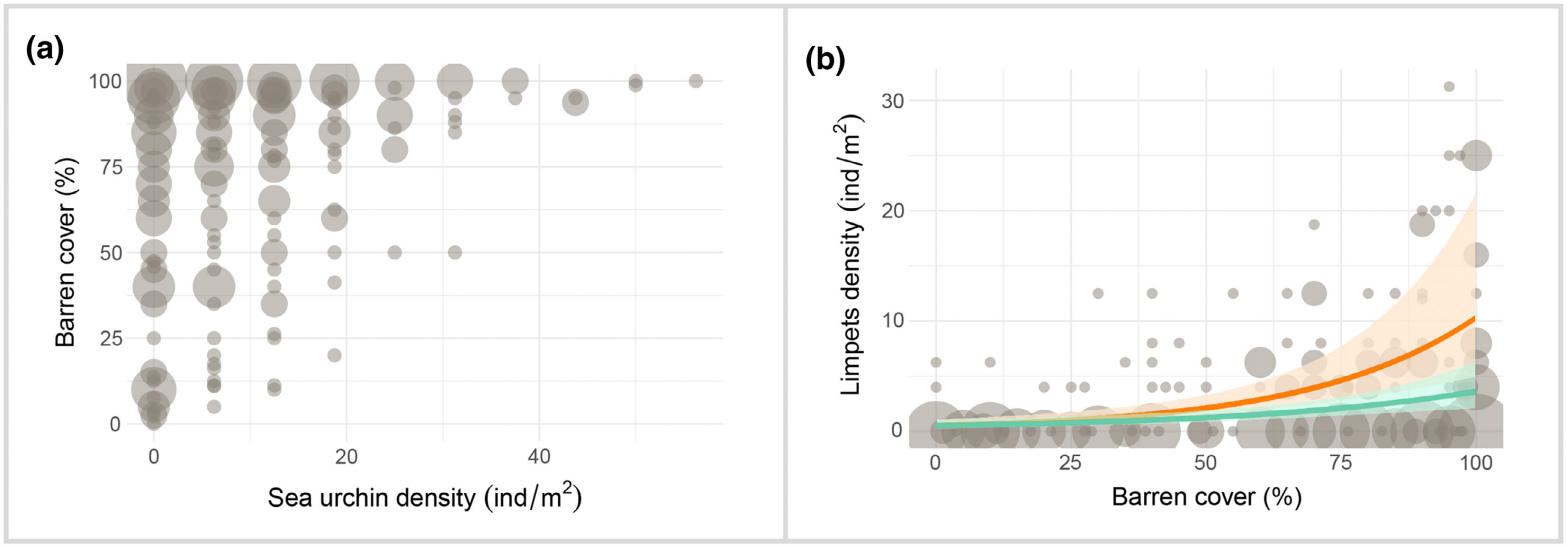
(a) Sea urchin density and barren cover. Bubble plot with values for the combination of sea urchin density (ind/m^2^) and barren cover (%) under high‐ and low‐nutrient conditions. The size of the bubbles is proportional to the number of specific combinations between both variables in quadrats (data from high‐ and low‐nutrients conditions have been included; *n* = 289). (b) Barren cover and limpet density. Bubble plot and predicted values (according to GLMMs; solid line) plus confidence intervals (shaded gray area) for limpet density (ind/m^2^) to barren cover. The interaction between barren cover and nutrient regime is significant (*p‐*value < .001). Data from high‐ (orange trend, *n* = 125) and low‐ (green trend, *n* = 164) nutrient conditions are plotted together and predicted values come from independent models.

We found a positive relationship between barren cover and sea urchin densities. In particular, above a certain threshold (~20 ind/m^2^) of sea urchin density, algae were virtually absent (Figure 2a). Limpet density significantly increased with barren cover (Figure 2b, Table 1); this pattern was significant in both regions and slightly stronger in high‐nutrient regions (Figure 2b, respectively, Table 1).

**TABLE 1:** Barren cover determines limpet densities.

Response variable	Explanatory variable	*χ* ^2^	df	*p*‐value
Limpet density	Barren Cover	15.95	1	**<.01**
	Nutrient condition	0.89	1	.35
Limpet density (HN)	Barren Cover	4.37	1	**.04**
Limpet density (LN)	Barren Cover	9.81	1	**<.01**

*Note*: Statistical results for generalized mixed models to test the effect of barren cover on limpet abundance. First the effect of barren cover is tested considering all the data pooled and (barren cover and nutrient conditions as fixed factors). Then, the same analysis was performed for each nutrient regime dataset specifically.

Singificance reference for bold values is .05.

### 3.2 Limpet grazing impact

**FIGURE 3:**
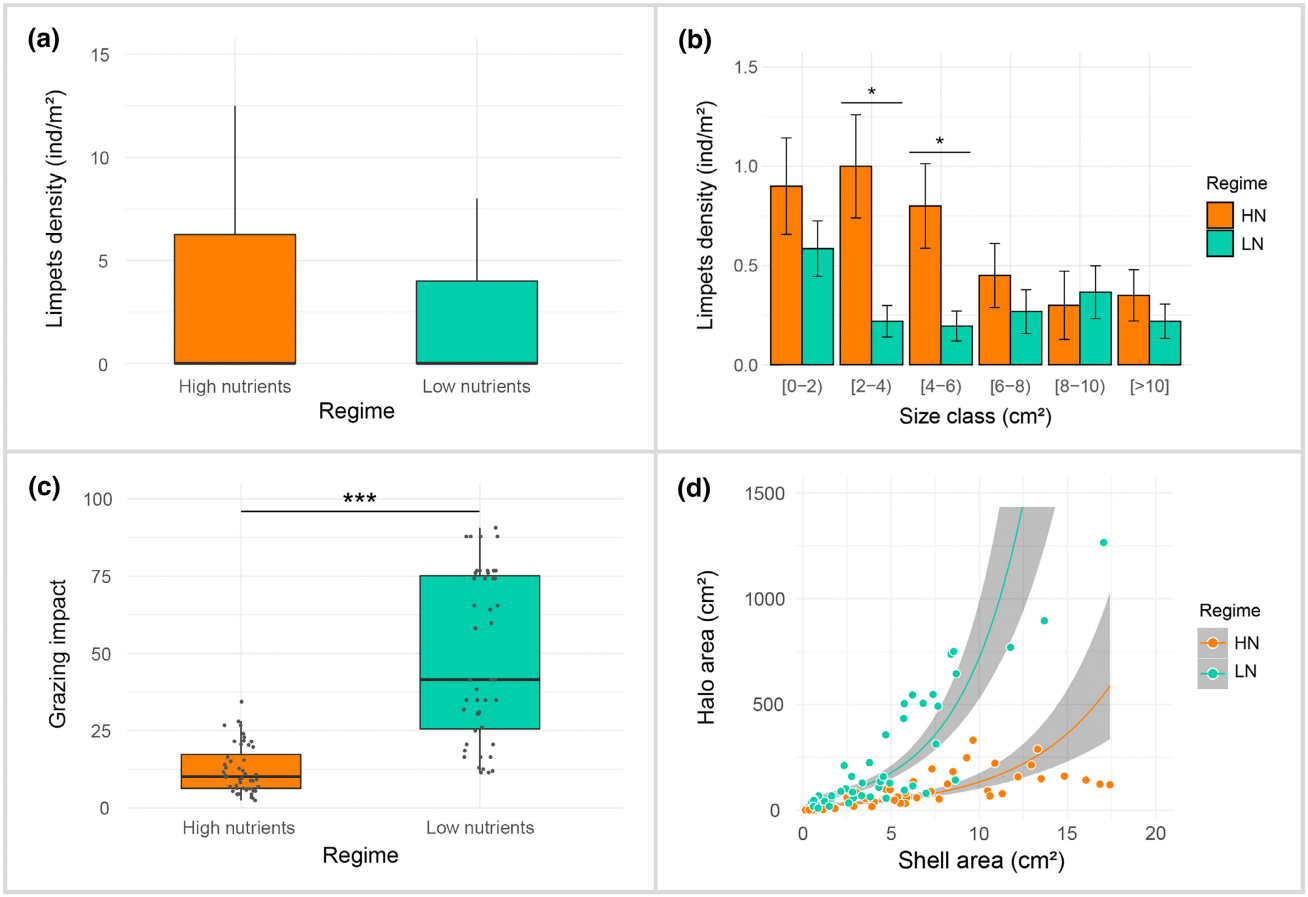
Effect of nutrient regime on limpet densities and grazing impact. (a) Box plots of the overall limpet densities (ind/m^2^ ± SE) presented in high‐ and low‐nutrient conditions. (b) Bar plot of mean limpet densities (ind/m^2^ ± SE) per size class in each nutrient condition. Significant differences are denoted by the symbol * (*p‐*value < .05). (c) Box plots depicting the effect of nutrient conditions on limpets grazing impact (ratio between halos and limpet shell areas) under high‐ (*n* = 48) and low‐ (*n* = 47) nutrient conditions, respectively. Significant differences are denoted by the *** (*p*‐value < .001). (d) Relationship between halos and limpet shell areas (cm^2^) for both nutrient conditions. Predicted values (solid line) and confidence interval (shaded area) are also shown. A significant trend between these variables is seen under both regimes (*p‐*value < .001).

Nutrient conditions regulate the impact of limpets grazing on macroalgae (Figure 3). Limpet density was slightly greater (non‐significant) in high‐ than in low‐nutrient regions (mean ind/m^2^ ± SE = 3.9 ± 0.62 vs. 1.88 ± 0.3, Table 1), mostly as a result of increased densities of limpets in size ranges of 2–4 and 4–6 cm^2^ (Figure 3a,b, Table 2). Also, the grazing impact of limpets varied significantly in accordance with nutrient conditions (Figure 3c, Table 3). The ratio between halo and limpet size was significantly smaller at high‐ compared to low‐nutrient regions (mean ± SE = 12.46 ± 1.12 versus 48.43 ± 3.88), suggesting that limpets with the same shell size produced larger halos in low‐nutrient conditions. The overall estimated grazing impact on the macroalgae at the population level was 4.7 times stronger at low‐nutrient conditions despite showing lower limpet abundances (that is, the sum of the mean grazing impact per size class multiplied by the average density in that size class). In both nutrient conditions, the size of halos was clearly related to shell area following an allometric relationship, with larger limpets causing a greater grazing impact than smaller individuals (Figure 3d, Table 4).

**TABLE 2:** Limpet densities at different nutrient conditions.

Response variable	Explanatory variable	*χ* ^2^	df	*p*‐value
Limpets Density	Nutrient condition	3.802	1	.051
Limpet size [0–2]	Nutrient condition	0.219	1	.639
Limpet size [2–4]	Nutrient condition	4.821	1	**.028**
Limpet size [4–6]	Nutrient condition	3.904	1	**.048**
Limpet size [6–8]	Nutrient condition	0.328	1	.566
Limpet size [8–10]	Nutrient condition	0.043	1	.834
Limpet size [>10]	Nutrient condition	0.775	1	.378

*Note*: Statistical results for generalized mixed models to test the effect of nutrient conditions in limpet densities altogether and per each size class.

Singificance reference for bold values is .05.

**TABLE 3:** Grazing impact.

Response variable	Explanatory variable	*χ* ^2^	df	*p*‐value
Grazing impact	Nutrient condition	24.034	1	**<.01**

*Note*: Statistical results for generalized mixed models to test the effect of nutrient conditions on overall grazing impact.

Singificance reference for bold values is .05.

**TABLE 4:** Halo area.

Response variable	Explanatory variable	*χ* ^2^	df	*p*‐value
Halo area	Limpet size	69.491	1	**<.01**
	Nutrient condition	13.188	1	**<.01**

*Note*: Statistical results for generalized mixed models to test the effect of limpet size and nutrient conditions in the halo size.

Singificance reference for bold values is .05.

### 3.3 Sea urchin removal experiment

**FIGURE 4:**
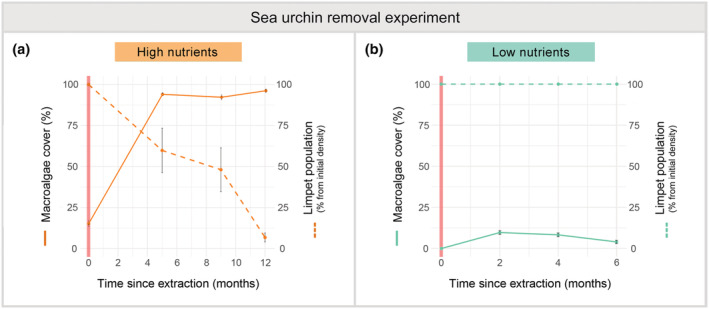
Effects of sea urchin removal under contrasting nutrient conditions. Changes in mean macroalgae cover, including both shrub‐forming and turf strata (solid lines and dots; %±SE), and in the percentage of limpet density (dashed lines and dots; % from initial density±SE) after removing sea urchins under (a) high‐ and (b) low‐nutrient conditions. Significant differences over time are seen for macroalgae cover and limpet population in a high‐nutrient regime (*p‐*value < .001). Pink shading indicates the time when sea urchins were removed from the selected barren areas.

At high‐nutrient conditions, we found a significant increase in the macroalgal cover 5 months after sea urchin removal (i.e., from 14.91% ± 1.82% to 93.67% ± 0.90%; mean ± SE, Figure 4a, Table 5). Macroalgal recovery was mainly due to early colonization of turf‐forming species, while algae with more complex morphology were rare. Thereafter, the macroalgae persisted at very high cover values throughout the experiment. In contrast, in control areas, macroalgal cover remained low 5 months after the start of the experiment (24.73% ± 4.13% mean ± SE). Limpet densities decreased following the recovery of macroalgae cover (Figure 4a, Table 5), from 3.9 ± 0.62 ind/m^2^ to virtually no individuals. In addition, the abundance of halos without limpets increased significantly (Figure S2).

**TABLE 5:** Two‐way mixed ANOVA results to test the significance in the recovery of macroalgal cover and decrease in limpet density in sea urchin removal experiments at both high‐ and low‐nutrient conditions. Model for limpet density in the low‐nutrient condition was not performed.

Nutrient condition	Response variable	Explanatory variable	*χ* ^2^	df	*p*‐value	Post hoc
High nutrients	Algal cover	Time	3715.8	3	**<.01**	T0 < T1 = T2 = T3
	Limpet density	Time	2784	3	**<.01**	T0 > T1 > T2 > T3
Low nutrients	Algal cover	Time	44.118	3	**<.01**	T0 = T1 = T2 > T3
	Limpet density	Time	—	—	—	—

Singificance reference for bold values is .05.

In contrast, under low‐nutrient conditions, the barren cover did not change 6 months after sea urchin removal for both treatment and control areas (Figure 4b, Table 5). Likewise, limpet densities showed negligible fluctuations throughout the experiment.

## 4 DISCUSSION

**FIGURE 5:**
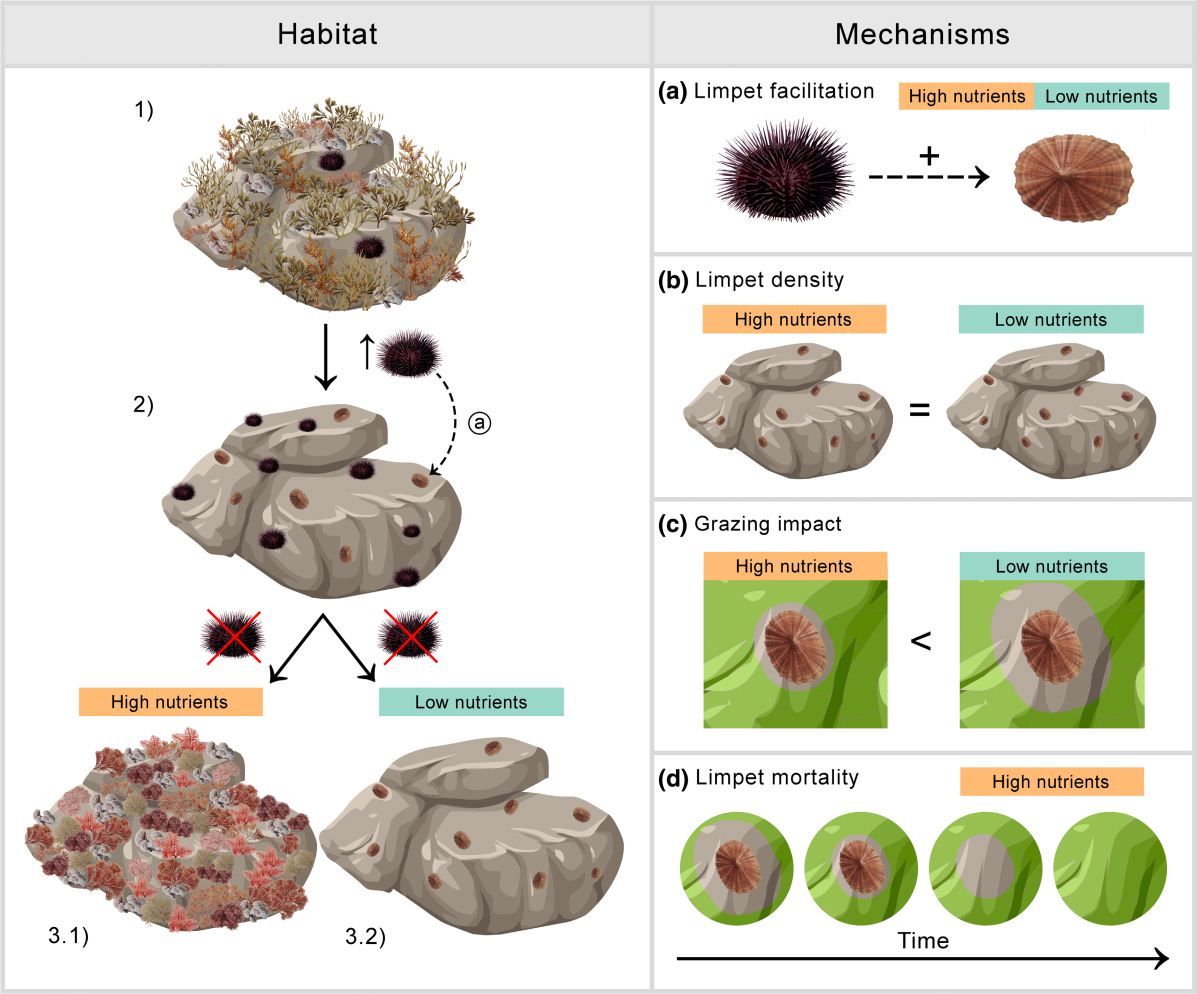
Diagram of the main findings explaining the dependence of reinforcing feedbacks to nutrient conditions. On the left, sea urchin overgrazing facilitates the presence of limpets by grazing on the macroalgal forests (1) and precipitating the formation of barrens (2). Differences in nutrient conditions determine the capacity of limpets to maintain barrens. In high‐nutrient conditions, limpets do not outcompete algae growth and turfs colonize the rock surface (3.1). Instead, in low‐nutrient conditions, limpet grazing is sufficient to maintain the barren state (3.2). On the right, the mechanisms underlying this feedback in both nutrient conditions are detailed (a) sea urchin facilitation of limpets, (b) slightly different limpet densities between nutrient regimes, (c) differential grazing impact according to nutrient conditions, and (d) increased limpet mortality related to the recovery of the macroalgae.

Environmental features set the essential conditions that define species interactions and ecosystem behaviors. Our findings show that abiotic differences in the environment (i.e., nutrient conditions) strongly regulate the onset of ecological feedbacks that maintain degraded ecosystem states in rocky reefs. The presence of limpets in rocky reefs is facilitated by sea urchin overgrazing and appears to act as a strong reinforcing mechanism for barren persistence, under low‐nutrient conditions. Their impact on macroalgal cover varies dramatically with the immanent nutrient conditions of a region, something that previous studies have already described (see Piazzi et al., 2016; Pinna et al., 2020). Under low‐nutrient conditions, these mesograzers can preclude macroalgal recovery in barren areas, even in the absence of intense consumer pressure by sea urchins (Figure 5). This proofs the prevalence of limpet grazing as critical feedback increasing the hysteresis of barren formation in oligotrophic waters and could explain the stability of large barrens along the coasts of the Eastern Mediterranean (Sala et al., 2012). In contrast, at high‐nutrient conditions, limpets are unable to sustain the persistence of barrens. While limpets may not trigger the formation of barrens, they certainly help maintain them, if the environmental conditions are favorable.

Regime shift dynamics are led by a destabilization of a given ecosystem state. From bushfires in the Serengeti‐Mara, to nutrient loads in estuaries and mountain lakes to unbalances in herbivory pressure in coral reefs and rocky reefs, changes in the levels of a given stressor promote the transition between alternative stable states (Dublin et al., 1990; Hughes, 1994; Knowlton, 1992; Lindegren et al., 2012; Petersen et al., 2008). Unbalances at any level of trophic organization could act as a stressor. The loss of top predators has unexpected effects on different taxa and ecological processes (Ripple et al., 2014). As seen in the Aleutian Islands, the removal of sea otters released the predation on herbivorous invertebrates and caused the collapse of kelp forests (Estes & Palmisano, 1974). In many temperate seas, like in our study case, grazing generated by high densities of sea urchins is the cause of marine forests shifting to barren habitats (Bulleri et al., 1999; Filbee‐Dexter & Scheibling, 2014; Ling et al., 2015; Pinnegar et al., 2000). The way the ecosystem reorganizes after shifting to an alternate state, defines the stability of the new state. For instance, in the Serengeti‐Mara, although the transition from woodlands to grasslands was not caused by elephants grazing, their presence was key to hold this new state. Therefore, elephants act as stabilizing feedback of the alternative stable state (Dublin et al., 1990). Unproductive barrens, very similar across regions and dominated by crustose coralline algae, represent an impoverished ecosystem state in shallow subtidal zones. This state promotes the colonization of benthic invertebrates, including limpets that further graze upon the rocks (Figure 5a; Pinnegar et al., 2000; Scheibling et al., 2009). Interestingly, our results underpin the importance of environmental conditions in determining the prevalence of ecological feedbacks and supports the idea that persistent regime shifts are determined by both biotic and abiotic conditions (see Conversi et al., 2015).

The barer the rock, the more limpets, but the ability of limpet grazing to maintain rocky reefs in a barren state depends on nutrient levels. Specifically, grazing marks (i.e., halo areas, strongly related to limpet size) were always smaller under high‐ than under low‐nutrient levels (Figure 5c). Our results are aligned to an earlier study on barren formation and stability in regions characterized by different nutrient conditions that confirmed the link between the ability of sea urchins to prevent the recovery of shrub‐forming macroalgae to an increase in consumption rates when plants are of low nutritional quality and to a decrease in algal growth rate, under nutrient limitation (see Boada et al., 2017). Limpet grazing capacity at low‐nutrient levels was, in fact, 5‐fold greater than that at high‐nutrient conditions, despite slightly smaller limpet densities.

At high‐nutrient conditions, algal growth exceeded consumption rates and thus, ephemeral macroalgae could recover within a relatively short period of time (i.e., 5 months). The removal of sea urchins from barrens in the high‐nutrient regions prompted the proliferation of fast‐growing turfing species that accounted for most of the algal recovery and caused a decrease in limpet abundance. Indeed, limpet density decreased significantly with time after sea urchin removal, along with an increase in the presence of halos without limpets (proxy of limpet mortality, Figure 5d; Figure S2). Reduced limpet survival seems to be a combination of a reduced foraging capacity due to the swamping by macroalgae (e.g., epilithic biofilm, Underwood & Jernakoff, 1981) and, hence, movement limitations (Jenkins & Hartnoll, 2001).

The results of our study show that limpet grazing acts as reinforcing feedback after sea urchin barrens are removed. However, this is only valid under limited nutrient conditions, where the slower growth of macroalgae together with the higher limpet grazing activity promotes barren stability. Instead, in the nutrient‐rich sites, turf and other macroalgae rapidly replenish barrens, after sea urchins are removed, breaking down the limpet feedback capacity and contributing to algal recovery. We acknowledge that further studies in other temperate nutrient‐rich regions may be essential to confirm the generality of this feedback in barrens worldwide. In the extreme oligotrophic Eastern Mediterranean, several studies have identified the persistence of barrens even after herbivory pressure (e.g., invasive species or sea urchin grazing) was reduced (Sala et al., 2011; Vergés, Tomas, et al., 2014). Although comparisons between Eastern and Western Mediterranean have to be made cautiously, since invasive herbivores in the Eastern Mediterranean make the composition of grazers very different between the two regions, we believe that the feedback identified in this study can be one of the major reasons for barren stability in some rocky reefs in the Eastern Mediterranean.

Earlier works highlighted the need to reduce consumption by herbivores (i.e., sea urchins) below a critical threshold to facilitate the recovery of marine forest (Boada et al., 2017; Bulleri et al., 1999; Pinna et al., 2020; Pinnegar et al., 2000). Indeed, sea urchin removal (culling or fishing) has been proposed in different regions where large predators are depleted as a strategy to promote the recovery of marine forests (Piazzi & Ceccherelli, 2019; Sanderson et al., 2015; Watanuki et al., 2010). However, our results indicate that these interventions may only work in some locations or more precisely under certain combinations of biotic and abiotic conditions since, as shown in our study, secondary mesograzers like limpets can maintain herbivore pressure high enough to prevent a shift back to the forested state under certain circumstances. Specifically, under low‐nutrient conditions, it is fundamental that these ecosystems do not exceed critical thresholds for the macroalgae, compromising the stability of the system given that the recovery will be always prevented by limpet mediated feedbacks.

Stochastic disturbances such as catastrophic storm events, as well as climate change, can have severe implications on the maintenance of the herbivory function in rocky macroalgal systems, leading to sea urchin mass mortalities (Hereu et al., 2012; Sanchez‐Vidal et al., 2012; Yeruham et al., 2015) as a result of their low mobility (Pagès et al., 2013). At the end of January 2020, an extreme storm event hit the Catalan coast (high‐nutrient region) causing a ~60% reduction of adult sea urchins in experimental control plots (personal observation). Interestingly, the macroalgal cover in these plots increased to levels comparable with those in sea urchin removal barrens. This event confirmed that our experimental results are a good approximation of what can happen in nature and indicate that similar events may have an important influence in shaping shallow rocky reef communities in a geographical context, depending on the environmental regimes.

Barrens and turfs are increasingly replacing marine forests as the dominant communities in temperate shores across the world (Filbee‐Dexter & Scheibling, 2014; Filbee‐Dexter & Wernberg, 2018; Pessarrodona et al., 2021) and shifts to less productive states may be very persistent in most cases (Filbee‐Dexter & Wernberg, 2018). Understanding the reasons for their persistence is the first step in arresting the spread of barrens and transforming them to productive marine forests. The primary destabilizing agents implicated in macrophyte decline are sea urchins, and the first challenge in habitat restoration is bringing their populations under control (Boada et al., 2017; Ling et al., 2015). However, it is important to recognize that each stability basin in systems characterized by alternate stable states is maintained by a unique set of feedbacks, sometimes with a completely different suite of interacting actors. These actors may not have been an important part of the healthy state, nor important to the collapse itself. Yet, in some environmental contexts, they may be critical to the alternate state and completely determine their hysteretic path (Filbee‐Dexter & Scheibling, 2014). Understanding these interactions well would make management strategies for these habitats much more effective and would help identify where simple interventions, like reducing sea urchin numbers, may be sufficient for habitat restoration as well as where additional interventions may be needed for recovery.

Our work highlights the importance of the interplay between trophic control, mediated by plant–herbivore interactions, and local environmental settings in shaping the structure, state and functionality of rocky reefs. Importantly, we demonstrate the capacity of environmental regimes to break down the capacity of ecological feedbacks to contribute to the stability of an ecosystem state and, hence, their prevalence in nature.

## AUTHOR CONTRIBUTIONS

**Laia Illa‐López:** Conceptualization (equal); data curation (equal); formal analysis (equal); investigation (equal); methodology (equal); visualization (equal); writing – original draft (equal); writing – review and editing (equal). **Àlex Aubach‐Masip:** Conceptualization (equal); data curation (equal); formal analysis (equal); methodology (equal); visualization (equal); writing – original draft (equal); writing – review and editing (equal). **Teresa Alcoverro:** Conceptualization (equal); data curation (equal); funding acquisition (equal); investigation (equal); methodology (equal); project administration (equal); visualization (equal); writing – original draft (equal); writing – review and editing (equal). **Giulia Ceccherelli:** Conceptualization (equal); data curation (equal); funding acquisition (equal); investigation (equal); methodology (equal); project administration (equal); writing – original draft (equal); writing – review and editing (equal). **Luigi Piazzi:** Conceptualization (equal); data curation (equal); methodology (equal); writing – original draft (equal); writing – review and editing (equal). **Periklis Kleitou:** Conceptualization (equal); investigation (equal); methodology (equal); writing – original draft (equal); writing – review and editing (equal). **Jorge Santamaría:** Conceptualization (equal); investigation (equal); methodology (equal); writing – original draft (equal); writing – review and editing (equal). **Jana Verdura:** Conceptualization (equal); investigation (equal); methodology (equal); writing – original draft (equal); writing – review and editing (equal). **Neus Sanmartí:** Conceptualization (equal); investigation (equal); methodology (equal); writing – original draft (equal); writing – review and editing (equal). **Elvira Mayol:** Conceptualization (equal); investigation (equal); methodology (equal); writing – original draft (equal); writing – review and editing (equal). **Xavi Buñuel:** Conceptualization (equal); investigation (equal); methodology (equal); writing – original draft (equal); writing – review and editing (equal). **Mario Minguito‐Frutos:** Conceptualization (equal); investigation (equal); methodology (equal); writing – original draft (equal); writing – review and editing (equal). **Fabio Bulleri:** Conceptualization (equal); investigation (equal); methodology (equal); writing – original draft (equal); writing – review and editing (equal).

## Supporting information


Figures S1–S2

## Data Availability

The data used in this study will be made publicly available at the Dryad open repository (https://datadryad.org/stash).
