# Supplementary material for: Nutrient conditions determine the strength of herbivore‐mediated stabilizing feedbacks in barrens
Source: Ecol Evol. 2023 Mar 21;13(3):e9929. doi: 10.1002/ece3.9929 (PMC10030269; doi:10.1002/ece3.9929)
Supplement: Supplementary file 1 — Figures S1–S2 [file ECE3-13-e9929-s001.docx]

**Supplementary:**


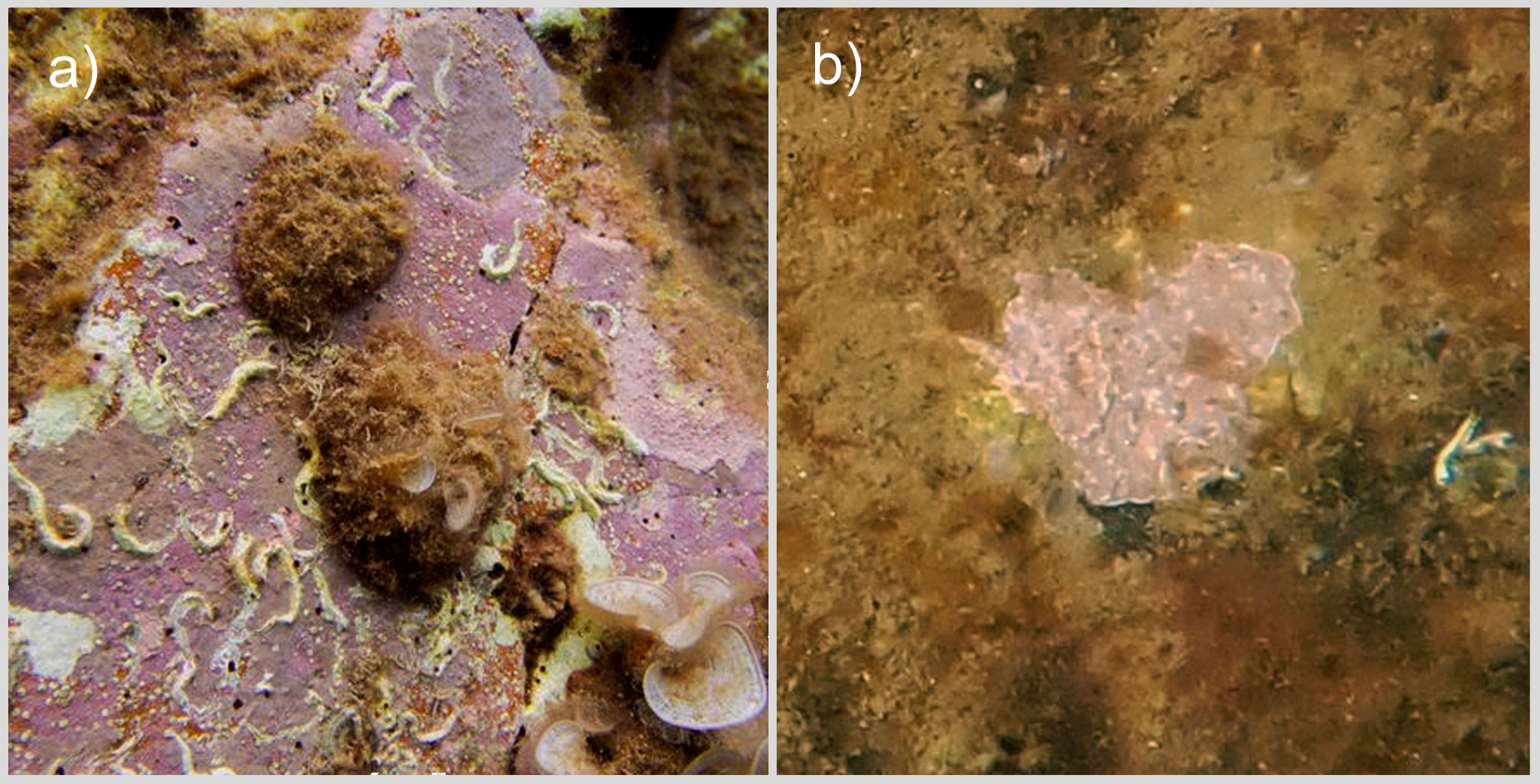


**Figure 1 | Limpet grazing marks. (a)** A halo generated by the grazing impact of two limpets. **(b)** A halo without the limpet that has created it.


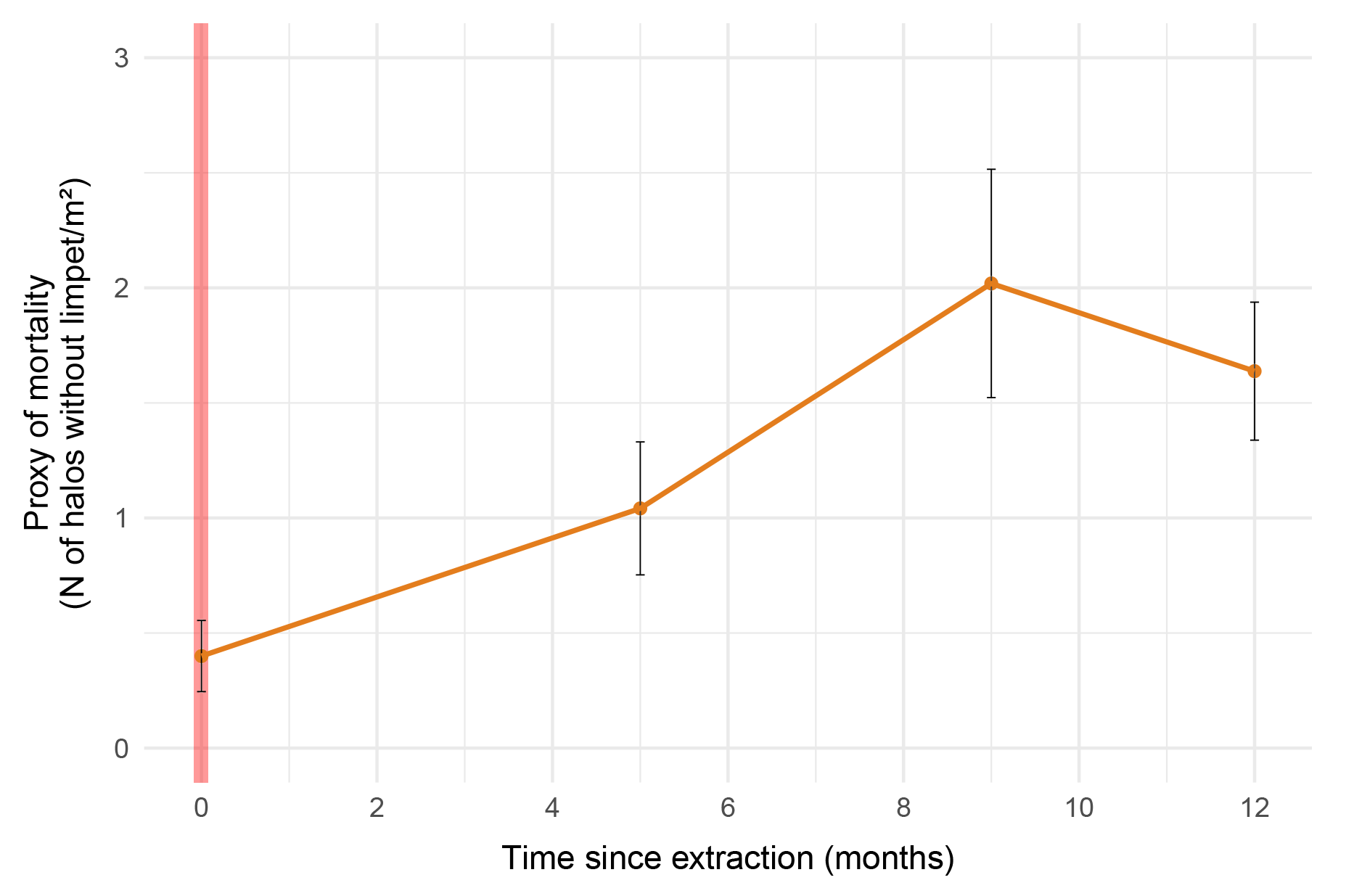


**Figure 2 | Evolution of halos without limpets during the experiment.** Changes in the mean number of halos without limpets per m^2^ (orange line and dots; mean ± SE) over time (months) after sea urchin removal. Differences in the number of halos without limpet along the experiment under high nutrient conditions were significant (*p-value* < 0.05).
